# Editorial: Innate lymphocytes in tumor surveillance

**DOI:** 10.3389/fimmu.2022.1093318

**Published:** 2022-11-23

**Authors:** Jörg H. Fritz, Dagmar Stoiber

**Affiliations:** ^1^ Department of Microbiology and Immunology, McGill University Research Center on Complex Traits (MRCCT), McGill University, Montreal, QC, Canada; ^2^ Department of Pharmacology, Physiology and Microbiology, Division Pharmacology, Karl Landsteiner University of Health Sciences, Krems, Austria

**Keywords:** innate immunity, natural killer cells, innate lymphoid cells, tumor surveillance, immunotherapy

The multifaceted roles of Innate Lymphoid Cells (ILC) have been widely interrogated in tumor immunity. Whereas Natural Killer (NK) cells possess tumor-suppressive properties across multiple types of cancer, the other ILC family members can either promote or inhibit tumor growth depending on the environmental conditions. The differential effects of ILCs on tumor outcome have been attributed to the high degree of heterogeneity and plasticity within the ILC family members. However, it is now becoming clear that ILC responses are shaped by their dynamic crosstalk with the different components of the tumor microenvironment (TME) (1). Recent years have witnessed a significant development in the current understanding of ILCs and their roles in the innate immune system, where they regulate tissue homeostasis, inflammation, as well as tumor surveillance and tumorigenesis (2). ILCs may be classified into three subgroups depending on their phenotypic and functional characteristics: Group 1 ILCs, which include NK cells and ILC1s (3); Group 2 ILCs, which only contain ILC2s (4), and Group 3 ILCs, which comprise of LTi cells and ILC3s (5).

This Research Topic features several review and original research articles on the different facets of innate lymphocytes in the context of cancer. This includes basic underlying mechanisms of anti-tumor action as well as translational and clinical advances in this area of research. The topic spans different areas of NK cell as well as ILC research with emphasis on their role during tumor development and progression.

NK cells are the prototype innate lymphoid cells exhibiting potent cytolytic function that provide host defense against infection and tumors. They are able to kill tumor cells if these show surface markers associated with oncogenic transformation. Due to this property NK cells control tumor growth at least in the early phase of tumor development and thus they are essential in tumor surveillance. Once target cells are recognized the balance of activating and inhibitory receptor signaling regulates their effector function against tumor targets (6). These properties and their capacity to enhance antibody and T cell responses highlight the role for NK cells as anticancer agents (7, 8). Current research is focused on investigating how NK cells may be manipulated and employed as therapeutic strategies for the treatment of cancer.

NK cells are of utmost importance in host protection during tumor development. Li and O´Sullivan review the spatiotemporal dynamics of host factors (tissue-specific and systemic) that lead to progressive dysfunction of NK cells while a tumor progresses. These include heterogeneous tumor architecture, temporal disease states, diverse cellular subsets in the microenvironment and the complex changes in NK cell states in response to all these factors. Understanding of these different signals that NK cells are confronted with may help identify new therapeutic targets to increase effectiveness of NK cell therapy for cancer.

Based on the underlying mechanisms of tumor cell evasion from NK cells a rationale for improvement of NK cell-based cancer immunotherapies is presented by Seliger and Koehl. This review highlights different factors contributing to immune escape or immunosurveillance by NK cells as well as benefits and limitations in clinical NK cell-based immunotherapies such as adoptive cell transfer-based approaches and engineered NK cell-based immunotherapies. Supported by preclinical and clinical studies additional possibilities for NK cells in cancer patient treatment are suggested *via* combination therapies, potentially leading to further clinical advances.

Tumino et al. focus on polymorphonuclear myeloid-derived suppressor cells (PMN-MDSCs) and report on their strong suppressive effect on NK cell numbers and anti-tumor activity in patients with primary or metastatic lung tumors. By complementing *in vitro* cell culture experiments the authors revealed that exosomes derived from PMN-MDSCs are responsible for a reduced NK cell-mediated anti-tumor activity and suggest potential for PMN-MDSCs as prognostic marker for clinical outcome.

Concentrating on the underlying signaling pathways of tumor-NK cell interactions Witalisz-Siepracka et al. review the different roles of JAK/STAT3 signaling in the dynamic interplay between NK and tumor cells. The authors include literature on how tumor cell-intrinsic STAT3 drives evasion from NK cells but also how STAT3 may regulate NK cell cytotoxicity, cytokine production and anti-tumor responses *in vivo*.

As mentioned above multiple approaches are being investigated to relieve NK cell immunosuppression in the tumor microenvironment. Immune checkpoint blockade inhibiting the programmed death-1 (PD-1)/programmed death-ligand 1 (PD-L1) axis seems not only acting on T cells but also on NK cells. However, the role of NK cells in PD-1/PD-L1 context is still a matter of debate (9). del Rio et al. aimed to address this topic and present data from a mouse xenograft model suggesting that NK cell anti-tumor function is independent of PD-L1 expression on A20 tumor cells.

ILCs regulate immune responses by responding and integrating diverse signals within local microenvironments and as such are ideally suited to sense malignant transformation and regulate tumor immunity. However, as ILCs have been associated with anti-tumor and pro-tumor activities, they have been suggested to exert dual functions during carcinogenesis by promoting or suppressing the malignant outgrowth of premalignant lesions. Warner et al. discusses emerging evidence that shows that ILCs can impact early tumor development by regulating immune responses against transformed cells, as well as the environmental cues that influence ILC activation in premalignant lesions.

Hepatocellular carcinoma (HCC) is one of the deadliest cancers worldwide. However, the role of the ILCs in HCC is still not well defined. Bouyarou and Golub provide an overview of the known roles and actions of ILCs in HCC with an emphasis on the importance of diverse signaling pathways in the tuning of their responses.

ILCs are preferentially enriched in barrier tissues such as the skin, with emerging evidence indicating their role in the control of melanoma. Wagner and Koyasu review the current understanding of ILCs role as the first line of defence against melanoma development and progression and discuss the possibility to harness their therapeutic potential.

ILC2s are important mediators of type 2 immunity and play important roles in allergic diseases, helminth infections, and tissue fibrosis (10). Studies over the past decade have reported that ILC2s exert context-dependent roles in cancer. Wu et al. review the roles of ILC2s in solid tumors and propose that ILC2s could serve as a predictor for tumor prognosis and a new therapeutic target after immunotherapy resistance.

Despite the growing body of research on ILCs, there are still plenty of knowledge gaps of how these cells are shaped by disease- and tissue-specific cues. This Research Topic features several articles that highlight our current knowledge about ILCs in tumor surveillance, pinpoint gaps in our understanding and discuss the possibilities to harness their therapeutic potential.

## Author contributions

All authors listed have made a substantial, direct, and intellectual contribution to the work and approved it for publication.

## Conflict of interest

The authors declare that the research was conducted in the absence of any commercial or financial relationships that could be construed as a potential conflict of interest.

## Publisher’s note

All claims expressed in this article are solely those of the authors and do not necessarily represent those of their affiliated organizations, or those of the publisher, the editors and the reviewers. Any product that may be evaluated in this article, or claim that may be made by its manufacturer, is not guaranteed or endorsed by the publisher.
